# Achilles tendon ruptures: still very much in the limelight, and still controversial. Not all research is as glittering as it may look

**DOI:** 10.1186/s13018-026-06685-8

**Published:** 2026-03-01

**Authors:** Filippo Spiezia, Francesco Oliva, Filippo Migliorini, Nicola Maffulli

**Affiliations:** 1 Department of Health Sciences, University of Basilicata, Viale Ateneo Lucano 10, 85100 Potenza, Basilicata, Italy; 2 Department of Orthopaedic and Trauma Surgery, Ospedale San Carlo, Via Potito Petrone, 85100 Potenza, Italy; 3 Department of Sports Traumatology, Universita’ Telematica San Raffaele, Via di Val Cannuta, 247, Roma, Italy; 4 Department of Trauma and Reconstructive Surgery, University Hospital of Halle, Martin-Luther University Halle-Wittenberg, Ernst-Grube-Street 40, 06097 Halle (Saale), Germany; 5 Department of Orthopaedic and Trauma Surgery, Academic Hospital of Bolzano (SABES-ASDAA), via Lorenz Böhler 5, 39100 Bolzano, Italy; 6 Department of Life Sciences, Health, and Health Professions, Link Campus University, Via del Casale di San Pio V, 00165 Rome, Italy; 7 Department of Trauma and Orthopaedic Surgery, Faculty of Medicine and Psychology, University La Sapienza, 00185 Rome, Italy; 8 Centre for Sports and Exercise Medicine, Barts and the London School of Medicine and Dentistry, Mile End Hospital, Queen Mary University of London, London, E1 4DG England; 9 School of Pharmacy and Bioengineering, Keele University School of Medicine, Stoke on Trents, ST4 7QB UK

## Abstract

Recent literature has renewed the never-ending interest in the management of Achilles tendon ruptures, but some articles have demonstrated inaccuracies in the interpretation and citation of original studies. We aims to clarify specific methodological and technical misconceptions regarding tendon grafting techniques and surgical positioning. The introduction of artificial intelligence in Achilles tendon surgery and rehabilitation may enhance diagnostic precision and patient-specific care while underlining the importance of preserving clinical reasoning, ethical transparency, and procedural competence. Accurate scholarship and critical appraisal remain essential to ensure scientific reliability and responsible innovation.

## Misinterpretation of Achilles tendon studies

There has been recent great interest in the management of Achilles tendon tears, with at times controversial results and recommendations [1]. We are seeing more patients with such injuries, and it is inevitable that, regardless of the management chosen, complications will arise. In some instances, however, recent articles published in high-impact journals may well raise more questions than answers, especially when the scientific literature is misinterpreted and misrepresented.

For example, in the article “Revision Surgery for Achilles Tendon Rupture: A Comprehensive Review of Treatment Options, Outcomes, and Complications and the Role of Artificial Intelligence” [2], reference 13 [3] refers to reconstruction for chronic tears of the Achilles tendon, not to revision surgery for Achilles tendon ruptures. In Section 4.5 of that article, the authors state that autologous semitendinosus and gracilis autografts require harvesting in the supine position. This is not what was described in the original article reporting the use of gracilis for chronic tears of the Achilles tendon [4], in which it was clearly stated that the harvest took place with the patient prone through a vertical 2.0–2.5 cm longitudinal incision over the pes anserinus. A similar technique was described when harvesting the semitendinosus tendon [5]: again, the tendon of the semitendinosus is harvested through a vertical, 2.5–3 cm longitudinal incision over the pes anserinus, with the patient prone. One of the authors of the index article [2] was an author of article [5], and it is unclear how these authors would have allowed such a fundamental error to occur.

We described the original technique of harvesting the tendons of gracilis and/or semtitendinosus from the pes anserinus with the patients prone. We realised that, in such position, harvesting the tendons could have been demanding, as the anatomy was ‘upside down’. We described in [3], duly cited in [2] as reference 13, how to harvest the semitendinosus tendon from the popliteal fossa, with the patient prone. Some surgeons may have chosen to harvest the tendons from the pes anserinus with the patient supine, and then, repositioned them prone to proceed with Achilles tendon reconstruction. Obviously, this would prolong the procedure, and make it more demanding. However, we never advocated this.

## Philosophical and ethical issues regarding AI in Achilles tendon surgery

Subheading “4.8. The Role of Artificial Intelligence” gives a deep analytic description of possible new applications of artificial intelligence (AI) in Achilles tendon surgery and rehabilitation. We posit that AI has potential limitations and risks when over-used in surgical contexts. In the right hands, AI may enhance diagnostic accuracy, and allow personalised patient management, accurate prediction of patient outcomes, and optimisation of surgical procedures. Nevertheless, AI carries the risk of diminishing the surgeon’s decision-making capabilities [6]. Over-reliance on AI carries the danger of favouring purely algorithmic outputs over surgeons’ and clinicians’ clinical judgment, weakening surgeons’ critical thinking and the courage to override AI recommendations, speckled by a “black-box” effect that makes it opaque how and where conclusions are deduced from [7].

AI decision-making may also raise ethical concerns, especially regarding informed consent, as patients may not fully understand how AI-driven decision-making relates to their disease and how indications for surgery are determined [7].

Excessive use of AI in surgical procedures may lead to deskilling of the hand and mind [8]. Notwithstanding the difficulty of employing AI in non-permissive environments (for example, in combat areas or low-income country settings), a system malfunction may occur from malware infection, cyber-attack, or unpredictable technical issues. Should surgeons not be able to perform “fly by wire” operations, they may not be able to take charge of the situation, with potentially major morbidity to the patient.

Academic training centres may integrate AI surgical-based programs with traditional knowledge and training, and no doubt surgeons in training may benefit from it. However, surgeons’ training should aim to form doctors capable of using intelligence and applying it properly to sick patients. To do this, it may not be enough simply to combine and extract data from the past to apply them to the present.

## Not all research is as glittering as it may look

A recent systematic review and meta-analysis reports on reconstruction procedures for chronic Achilles tendon ruptures [9].The Modified Coleman Score was used to assess the quality of the included studies. However, no relevant reference was given to the original article [10] nor to the article which subsequently applied the score to Achilles tendon rupture. A recent systematic review on the use of free tendon grafts for the surgical management of chronic tears of the main body of the Achilles tendon concluded that, when faced with such chronic injuries with a gap of at least 6 cm, free tendon grafts allow predictable return to sport and acceptable recovery of function [11]. In the same year, the literature on local tendon transfers for the same pathology [12] was also systematically reviewed: the use of local tendon transfer for chronic Achilles tendon ruptures, namely the tendon of flexor hallucis longus or of peroneus brevis, resulted in good clinical outcomes and a reliable return to daily activities and sports.

The original work on the use of free gracilis tendon graft for neglected tears of the Achilles tendon [4] was however not acknowledged. In that work, published in 2005, a total of 21 patients were followed for two years. Fifteen of those patients were part of the long-term study (10.9 years) reported in reference 21. of the article by Dr Karaismailoglu and coll.

Hendriks et al. [13] published one of the largest single-centre studies on the management of acute Achilles tendon tears using either open or minimally invasive techniques, confirming that modern minimally invasive repairs offer grossly equivalent outcomes [14], with reduced risk of iatrogenic sural nerve problems [15].

From Table 1 [13], it appears that the average time between injury and the index surgery was at most in the region of two weeks, and the authors rightly comment that future investigations should concentrate on the influence of time to surgery in terms of postoperative complications. A study [16] recruiting 21 consecutive patients who presented between 14 and 30 days after an acute Achilles tendon tear compared them with 21 patients who were matched according to sex, age (± 2 years), and level of activity, who presented within 14 days of the index injury. At 12 months after percutaneous repair under local anaesthesia, both patient groups had a median Achilles tendon rupture score of 91. There were no significant differences between groups in terms of mean Achilles tendon resting angle. No patient in either group developed a wound infection. One patient in the acute group experienced an iatrogenic sural nerve injury. Achilles tendon rupture treated by percutaneous repair 14 to 30 days after injury achieved results at 1 year similar to those of patients treated < 14 days after injury.

It appears that, despite the widely available and easily searchable databases, the details of some articles can still be ignored and misinterpreted. In these instances, errors can be introduced into the scientific literature and perpetuated indefinitely.

## Data Availability

No datasets were generated or analysed during the current study.
